# Cultivation of Piezotolerant and Piezophilic Hyperthermophiles with a Newly Developed Constant High Pressure and Temperature Culturing and Monitoring System

**DOI:** 10.1264/jsme2.ME23055

**Published:** 2023-10-20

**Authors:** Fumiaki Mori, Akira Ijiri, Tomoya Nishimura, Taisuke Wakamatsu, Nozomi Katsuki, Yuki Morono

**Affiliations:** 1 Geomicrobiology Group, Kochi Institute for Core Sample Research, Extra-cutting-edge Science and Technology Avant-garde Research (X-star), Japan Agency for Earth-Marine Science and Technology (JAMSTEC), Monobe B200, Nankoku, Kochi 783–8502, Japan; 2 Graduate School of Maritime Sciences, Kobe University, 5–1–1 Fukaeminamimachi, Higashinada-ku, Kobe 658–0022, Japan; 3 Applied Science, Graduate School of Integrated Arts and Sciences, Kochi University, Monobe B200, Nankoku, Kochi 783–8502, Japan; 4 Agricultural Science, Graduate School of Integrated Arts and Sciences, Kochi University, Monobe B200, Nankoku, Kochi 783–8502, Japan; 5 Faculty of Life and Environmental Sciences, University of Tsukuba, 1–1–1 Tennodai, Tsukuba, Ibaraki 305–8572, Japan

**Keywords:** high hydrostatic pressure, high temperature, hyperthermophile, extremophile

## Abstract

The Earth’s microbial biosphere extends from ambient to extreme environments, including deep-sea hydrothermal vents and subseafloor habitats. Despite efforts to understand the physiological adaptations of these microbes, our knowledge is limited due to the technological challenges associated with reproducing *in situ* high temperature (HT)-high hydrostatic pressure (HHP) conditions and sampling HT-HHP cultures. In the present study, we developed a new high temperature and pressure (HTP) incubation system that enabled the maintenance of HT-HHP conditions while sampling incubation medium and mostly eliminated non-biological reactions, including hydrogen generation or the leakage of small gaseous molecules. The main characteristics of our system are (1) a chamber made of gold with gold-etched lid parts that suppress the majority of non-biological reactions, (2) the exceptional containment of dissolved gas, even small molecules, such as hydrogen, and (3) the sampling capacity of intra-chamber liquid without depressurization and the isobaric transfer of a culture to inoculate new medium. We initially confirmed the retention of dissolved hydrogen in the incubation container at 82°C and 20‍ ‍MPa for 9 days. Cultivation tests with an obligate hyperthermophilic piezophile (*Pyrococcus yayanosii*), hydrogenotrophic hyperthermophile (*Archaeoglobus profundus*), and heterotrophic hyperthermophile (*Pyrococcus horikoshii*) were successful based on growth monitoring and chemical ana­lyses. During HTP cultivation, we observed a difference in the duration of the lag phase of *P. horikoshii*, which indicated the potential effect of a pressure change on the physiology of piezophiles. The present results suggest the importance of a cultivation system designed and developed explicitly for HTP conditions with the capacity for sampling without depressurization of the entire system.

The ocean covers 70% of the Earth’s surface and hosts an enormous microbial population, spanning from the water column to underneath the seafloor. Whitman *et al.* (1998) reported that marine water contains an estimated 1.2×10^29^ cells, while the deep subseafloor harbors 2.9–5.4×10^29^ cells (Kallmeyer *et al.*, 2012; Parkes *et al.*, 2014; Bar-On *et al.*, 2018). These microbes live under various physical and chemical conditions. In the ocean, hydrostatic pressure increases linearly by 10 and 15 MPa per 1,000‍ ‍m in water and subseafloor sediment, respectively (Oger and Jebbar, 2010). Since approximately 89% of the ocean has a depth >1,000‍ ‍m (Costello *et al.*, 2010), most microbes dwelling in the deep sea and subseafloor endure high hydrostatic pressure (HHP) exceeding 10 MPa.

The deep sea is generally characterized by HHP and a low-temperature environment, within the range of 2–4°C. Although their number is relatively small given the broad area of a vast ocean, more than 300 deep-sea hydrothermal vents exist in the seafloor (Thornburg *et al.*, 2010) and release anoxic, chemical-rich, hot water (up to at least ~407°C, Koschinsky *et al.*, 2008; Dick, 2019). These hydrothermal vents are renowned for hosting rich communities of microbes and animals and are regarded as critical microbial habitats for investigating the conditions that limit microbial life (Takai *et al.*, 2008), the origin of life, and extraterrestrial life (German *et al.*, 2022). In addition to these vents, a high temperature (HT) environment extends under the seafloor. Temperature increases with sedimentary depth under the seafloor, with ~35% of the total volume of sediment exceeding 60°C (LaRowe *et al.*, 2017). A recent study demonstrated that microbial cells and their activities were detected in subsurface sediment up to 120°C (Heuer *et al.*, 2020; Beulig *et al.*, 2022). However, the upper-temperature limit for microbial life under the seafloor remains unclear. Therefore, further studies on the microbes inhabiting high temperature and high hydrostatic pressure (HT-HHP) conditions will provide critical information for understanding life and its full capacity.

HT and HHP affect the physiological properties of microbial life (Yang *et al.*, 2021). To investigate their diverse effects, various cultivation systems have been developed to examine the impact of HT-HHP on microbial cells (Kyo *et al.*, 1991; Nelson *et al.*, 1992; Tasumi *et al.*, 2017) (Oliver *et al.*, 2021 High temperature and high hydrostatic pressure cultivation, transfer, and filtration systems for investigating deep marine microorganisms. *Preprints*. https://doi.org/10.20944/preprints202104.0453.v1), and have provided insights into the physiological properties of (hyper) thermophiles and the geochemical responses of natural samples (Ohtomo *et al.*, 2013). Nevertheless, difficulties are associated with investigating the precise physiological properties of hyperthermophiles using HT-HHP systems because it is challenging to maintain consistent control over incubation conditions (*e.g.*, pressure, temperature, and nutrient concentrations, such as gaseous molecules).

We herein describe a newly developed cultivation system that is operatable at a pressure of up to 100 MPa and high temperature (up to 230°C). Our approach employs a gold cultivation tube, designed to minimize non-biological chemical reactions (mainly by water-metal reactions) and enable the exceptional containment of dissolved gases during cultivation. It is also possible to collect and add liquid medium during cultivations, thereby enabling the monitoring of temporal changes in cell abundance and chemical variables in a single cultivation tube without depressurization. Furthermore, isobaric medium transfer from one cultivation tube to another is conducted, thereby effectively minimizing the negative impact of depressurization and repressurization on microbial cells during inoculations. As a pilot study, we performed a cultivation experiment on an obligate hyperthermophilic piezophile (*Pyrococcus yayanosii*, Birrien *et al.*, 2011), piezotolerant hydrogenotrophic hyperthermophile (*Archaeoglobus profundus*, Burggraf *et al.*, 1990), and piezotolerant heterotrophic hyperthermophile (*Pyrococcus horikoshii*, González *et al.*, 1998). Based on the results obtained herein, we discussed the utility of this system, high-pressure microbial physiology, and its future application to natural samples, such as marine sediment.

## Materials and Methods

### Description of the HTP-Kochi core center (KCC) cultivation system

**Fig. 1.**
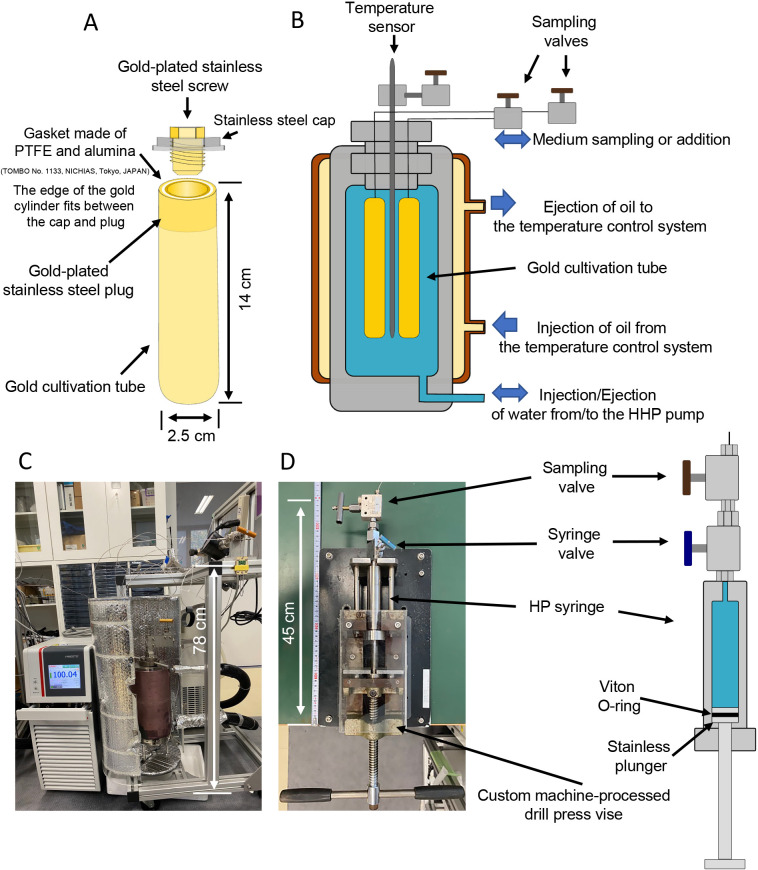
Schematic diagram and photograph of the HTP-KCC cultivation system. (A) The inner incubation chamber, (B) outer pressure chamber, (C) entire HTP-KCC cultivation system, and (D) HP syringe. Up to four inner incubation chambers can be placed in the outer pressure chamber. The interior volume of the inner incubation chambers is approximately 60‍ ‍mL. The system operates at pressures up to 100 MPa and 230°C.

The HTP-KCC cultivation system is composed of the following three parts:

(i) The inner incubation chamber is an end-closed gold tube (outer diameter: 2.5‍ ‍cm, height: 14.2‍ ‍cm, approximate interior volume: 60‍ ‍mL, Fig. 1A). The wall of this tube is thin (0.25‍ ‍mm), thereby allowing it to change its shape upon the introduction and sampling of the interior liquid. To minimize contact between medium and a reactive metal surface, the open end of the gold tube is sealed with a metal plug plated with gold. We also use a 1/16-inch gold/stainless steel double tube from the plug to the exterior valve to further minimize contact between medium and a reactive metal. The tube extends outside the outer pressure chamber, and the end of the tube is closed using an external valve (BuTech SLPV21, Haskel).

(ii) The outer pressure chamber (Fig. 1B and C), a pressure-resistant vessel (max. 100 MPa), has the capacity to hold four inner incubation chambers. The outer pressure chamber is filled with water, which applies and maintains hydrostatic pressure. This pressure is regulated by an external high-pressure syringe pump (65D syringe pump, Teledyne Isco). A temperature-controlling jacket is also attached to the chamber, allowing heat-transferring oil circulation through a temperature control system (PRESTO A30, Julabo).

(iii) A high-pressure syringe (Fig. 1D) is a compact pressure chamber equipped with a movable plunger and high-pressure valve (MVE1001, Autoclave Engineers). The syringe body is made of stainless steel and has an inner volume of approximately 7‍ ‍mL. It is connected to the external valve of the inner incubation chamber to add/collect liquid medium to/from inside the inner incubation chamber. The syringe is placed in a custom machine-processed drill press vise. When connected to the interior incubation chamber, the pressure of the entire system is kept constant while adding/collecting liquid medium by compensating the volume of water in the outer pressure chamber using an external high-pressure syringe pump. The valve disconnects the syringe while maintaining pressure, and sampled medium is transferred to another incubation chamber without depressurization.

### Cultivation test of hyperthermophilic archaea under HT-HHP conditions

Three hyperthermophilic archaea, *P. yayanosii* CH1^T^ (JCM 16557^T^), *A. profundus* AV18^T^ (JCM 9629^T^), and *P. horikoshii* OT3^T^ (JCM 9974^T^), were cultivated under HT-HHP conditions using the newly developed HTP-KCC cultivation system. These strains were provided by the Japan Collection of Microorganisms (JCM), RIKEN BRC, Japan. Culture media were prepared according to the growth medium specifications provided by JCM for each strain (JCM medium no. 214 for *A. profundus*, no. 151 for *P. horikosii*, and no. 811 for *P. yayanosii* on the website of the JCM On-line Catalogue Database, https://jcm.brc.riken.jp/en/). Incubation conditions for the two strains were set at their reported optimum temperatures and pressures: 98°C and 52 MPa for *P. yayanosii* CH1^T^ (Birrien *et al.*, 2011) and 82°C and 20 MPa for *A. profundus* AV18^T^ (Burggraf *et al.*, 1990). In the *A. profundus* AV18^T^ cultivation experiment, gaseous hydrogen was dissolved into medium in the pressure chamber at 20 MPa overnight to introduce hydrogen into the incubation chamber, transferred to the high-pressure syringe (HP syringe), and isobarically inoculated into the incubation chamber.

### Comparison of effects of pressure changes during inoculations on the growth rate

To assess the impact of pressure changes during inoculations on the growth of *P. horikoshii* OT3^T^, we prepared inocula that were pre-cultured under ambient or HHP conditions (20 MPa) and then inoculated them into the inner incubation chamber of the HTP-KCC system (20 MPa). A comparison was made with the ambient pressure incubation using an inoculum pre-cultured under ambient pressure conditions. In the pre-cultivation step, we conducted a conventional batch-type ambient pressure cultivation and HT-HHP cultivation (20 MPa) using the HTP-KCC system at 98°C for 13.5‍ ‍h. One milliliter of each pre-culture was then inoculated into the inner incubation chamber containing approximately 60‍ ‍mL of medium within the HTP-KCC system, which had already been set up at 98°C and 20 MPa. Alternatively, 0.5‍ ‍mL of each pre-culture was inoculated for ambient pressure cultivation into a 50-mL vial containing 30‍ ‍mL of medium.

### Monitoring of parameters: cell count, gas chromatography, and ion chromatography

Regarding cell counting, 100 or 500‍ ‍μL of the sampled medium was fixed with 1‍ ‍mL 10% (v/v) formalin and 3% (w/v) NaCl and stored at 4°C. Cell counting was conducted using a fluorescent staining method with epifluorescence microscopy and a flow cytometer described by Morono *et al.* (2009), Morono *et al.* (2013), and Mori *et al.* (2021). Some modifications were made for cell counting with flow cytometry based on Mori *et al.* (2021). In brief, 100‍ ‍μL of the fixed culture medium was mixed with 40‍ ‍μL of 1/250 diluted SYBR Green I (Thermo Fisher Scientific) in TE buffer and incubated for 30‍ ‍min in the dark. Subsequently, 660‍ ‍μL of TE buffer and 200‍ ‍μL of a custom-made suspension of fluorescence beads (Green: 505/515‍ ‍nm, Deep Red: 633/660‍ ‍nm, concentration: 2.66×10^5^ beads mL^–1^) were added. The sample was then analyzed using a Gallios flow cytometer (Beckman Coulter), following the procedure outlined in Mori *et al.* (2021). Cytogram settings for microbial cells in the logarithmic growth phase were used for cell detection, with adjustments for each microbial strain (Supplementary Fig. S1 and S2).

To measure the concentrations of dissolved gases, gas extraction from a liquid sample was performed as described by Ijiri *et al.* (2012) with slight modifications. Approximately 1‍ ‍mL of the sample was collected into a pre-evacuated glass container, to which 500‍ ‍μL of phosphoric acid solution was added. This transfer of medium from the HHP condition (at least 20 MPa) to the pre-evacuated glass, followed by acidification with the phosphate solution, facilitated the release of dissolved gases into the headspace of the glass container for gas sampling. Using gas chromatography, the extracted gas was analyzed for hydrogen, methane, and dissolved inorganic carbon (DIC) concentrations. Two gas chromatographs were employed: SRI 8610C (SRI Instruments) and Agilent 7890B GC (Agilent Technologies), equipped with a helium ionization detector.

The concentration of sulfate in the medium was measured using an ion chromatograph (Dionex ICS-2100, Thermo Fisher Scientific).

### Calculations and plotting of graphs

All calculations and plotting of graphs were performed with R‍ ‍(version 4.2.2, (R Core Team, 2022) using the “ggplot2” (Wickham, 2016) and “patchwork” (Pedersen, 2020) packages. To calculate the growth rates of each strain, linear fitting to observation data was performed by the lm function of R using at least three adjacent points in the logarithmic growth phase.

## Results and Discussion

### System performance check of the HTP-KCC cultivation system

While developing our HTP system, we aimed to construct a HHP incubation chamber that enables (i) the detection of biological reactions while minimizing the interference of non-biological reactions occurring under HT conditions, such as water-metal reactions, and (ii) isobaric injections and sampling. During previous attempts at HHP and/or HT cultivation, we encountered challenges related to non-biological changes in incubation environments, such as diffusion in/out of the incubation chamber and water-metal reactions. For example, if the incubation chamber is constructed from metal, such as stainless steel, there is a risk of chamber corrosion due to water-metal reactions (Nelson *et al.*, 1991), which may lead to the generation of hydrogen gas (Eba *et al.*, 2020). Although water-metal reactions have been utilized beneficially in the cultivation of anaerobic methanogens (Takai *et al.*, 2008), they may significantly affect the concentrations of dissolved gases and the overall integrity of incubation chambers. Tasumi *et al.* (2017) described an effective method for the high-pressure cultivation of anaerobic piezophiles, which involved using a glass vial attached to a glass-made syringe through a butyl rubber stopper. This incubation set-up does not allow any addition to or sampling from the incubation chamber. It was necessary to complete all the preparations, including inoculations, needed under ambient pressure conditions before pressurization or after depressurization. Due to these limitations, we aimed to develop a system that suppresses non-biological reactions and enables isobaric fluid transfer under already pressurized conditions. To achieve this, we utilized gold, either in its pure form or plated, on parts in direct contact with incubation medium. More detailed information is provided in the Materials and Methods section and Fig. 1.

To assess the performance of our newly developed system, we conducted a preliminary test using non-inoculated media (medium for *A. profundus*) that examined the effects of the absence of a water-metal reaction and the retention of gaseous compounds within our gold chamber. Hydrogen, methane, and dissolved inorganic carbon (DIC) concentrations were monitored over a 9-day incubation period at 82°C and 20 MPa, representing the optimal growth temperature and isolated water depth for *A. profundus* AV18^T^. Non-biological reactions that generate gaseous compounds were not observed throughout the incubation period, and the hydrogen and DIC concentrations added remained stable (Supplementary Fig. S3). In a separate experiment, we noted that hydrogen and methane diffused and leaked out from the incubation chamber sealed with a Viton O-ring (data not shown). To address this issue, we sealed the gold tube using a gold-plated stainless steel plug with a Teflon gasket (Fig. 1). While Viton rubber has a wide working temperature range (~230°C) and relatively low permeation coefficients for gases, including hydrogen, the complete closure of the gold chamber was necessary to retain all the components introduced into the incubation chamber.

Another critical issue associated with HT-HHP cultivation is the effects of depressurization on the physiology of pressure-incubated microbial cells. Previous studies reported that although depressurization did not kill piezophiles, repeated pressurization and depressurization cycles negatively impacted the growth rate and cell yield of these cells (Cario *et al.*, 2022). This finding highlights the importance of maintaining isobaric conditions throughout the incubation period. To avoid the negative effects of depressurization, some HT-HHP culture systems incorporate an external valve connecting to the cultivation chamber within the HT-HHP vessel. This type of design allows for the subsampling of liquid medium without depressurization. Previous studies employed this approach in their HT-HHP systems (Nelson *et al.*, 1992) (Oliver *et al.*, 2021 High temperature and high hydrostatic pressure cultivation, transfer, and filtration systems for investigating deep marine microorganisms. *Preprints*. https://doi.org/10.20944/preprints202104.0453.v1). The external valve enables the extraction of liquid samples without disturbing the pressure inside the chamber, thereby maintaining isobaric conditions. This technique is crucial for preserving the physiological state of cells during sampling and ensuring an accurate ana­lysis of their growth and metabolic activities. Our system is a modified version of the flexible cell-type reaction system described by Yoshizaki *et al.* (2009). The key feature of our system is the utilization of a flexible inner incubation chamber with confining pressure control. The inner incubation chamber is connected to a metal tube, which consists of a dual layer with a thin gold tube penetrating through the lid of the pressure chamber to the outside. This connection is sealed with high-pressure valves. We employ a HP syringe, which functions as a mini-pressure chamber, to retrieve liquid samples from the inner incubation chamber while maintaining isobaric conditions. The syringe pump controls the pressure inside the chamber to apply confining pressure (Fig. 1B). The use of the HP syringe allows us not only to collect media, but also to deliver new culture media or required compounds into the inner incubation chamber isobarically. Therefore, we have access to the inner chamber without opening the pressure chamber, thereby ensuring that pressure remains constant. This capability is useful for various tasks, such as taking time-series samples to monitor cell concentrations and dissolved chemical concentrations (including dissolved gases) in media. It also enables the supply of freshly prepared media and substrates into the chamber for long-term cultivation studies. To accurately monitor temperature, we inserted a temperature probe into the pressure chamber. This probe is positioned within a few millimeters of the interior gold incubation tube, thereby allowing precise monitoring of the temperature of the sample. The heating circulator, attached to the exterior heating jacket, uses the temperature probe as a target for maintaining the desired temperature during the experiment (Fig. 1B).

### Cultivation test of the HTP-KCC system for hyperthermophile archaea

We investigated the growth of two different strains: (i) *P. yayanosii* CH1^T^, an obligate piezophile requiring pressures higher than 50 MPa for optimal growth (Birrien *et al.*, 2011), and (ii) *A. profundus* AV18^T^, a piezotolerant strain that relies on hydrogen gas as an obligate electron donor (Burggraf *et al.*, 1990).

**Fig. 2.**
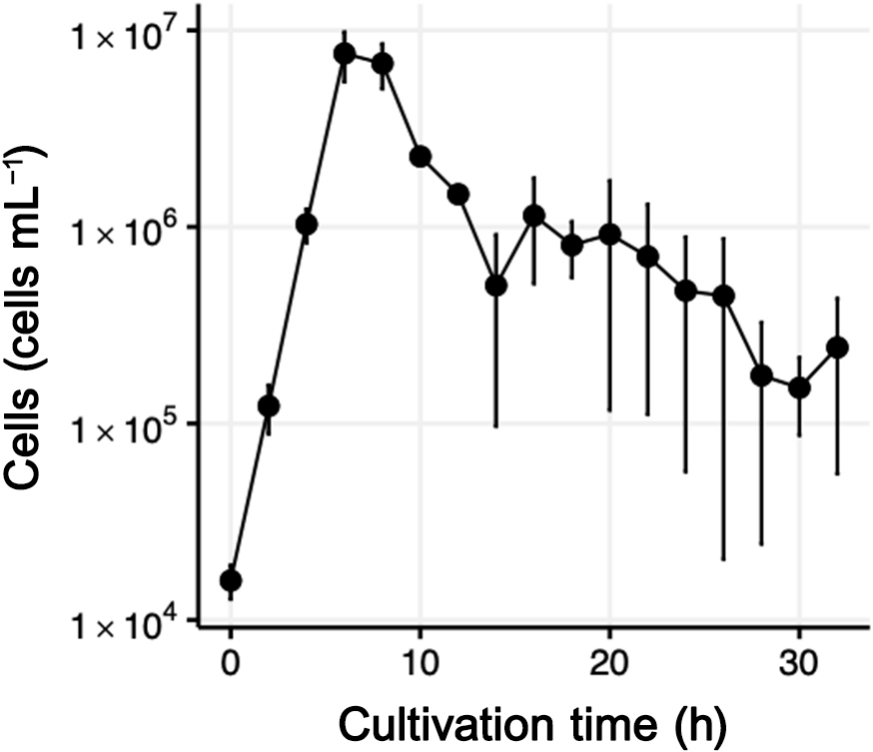
Growth curve of *Pyrococcus yayanosii* CH1^T^ during a cultivation experiment at 52 MPa and 98°C in the HTP-KCC system. Error bars represent the range of cell abundance observed in duplicate experiments.

Regarding *P. yayanosii* CH1^T^, we inoculated the pressure-incubated and ambient pressure-stored *P. yayanosii* CH1^T^ culture into medium in the inner incubation chamber after reaching the high hydrostatic pressure (52 MPa) and a temperature of 98°C. We monitored the growth of the culture every 2 h. The cell concentration reached a plateau of 7.6×10^6^‍ ‍cells‍ ‍mL^–1^ after 6‍ ‍h (Fig. 2). The maximum cell counts observed in our cultivation were lower than that reported in the literature (at the order of 10^8^, Birrien *et al.*, 2011). This difference may be attributed to our strict counting method using flow cytometry. In the flow cytogram of our culture, we identified particles with faint or diminished fluorescence (Supplementary Fig. S1). Upon microscopic observations, we also detected two types of cells with different fluorescence intensities. In our experiences of staining microbial cells with SYBR Green I (>500-fold increase in its quantum yield upon binding to DNA), we defined “cells” as those with bright fluorescence. We did not count particles with faint fluorescence. The observed doubling time of the culture was approximately 41‍ ‍min, which was slightly shorter than the previously reported doubling time of 50‍ ‍min (Birrien *et al.*, 2011), indirectly showing the appropriateness of our cell recognition criteria.

**Fig. 3.**
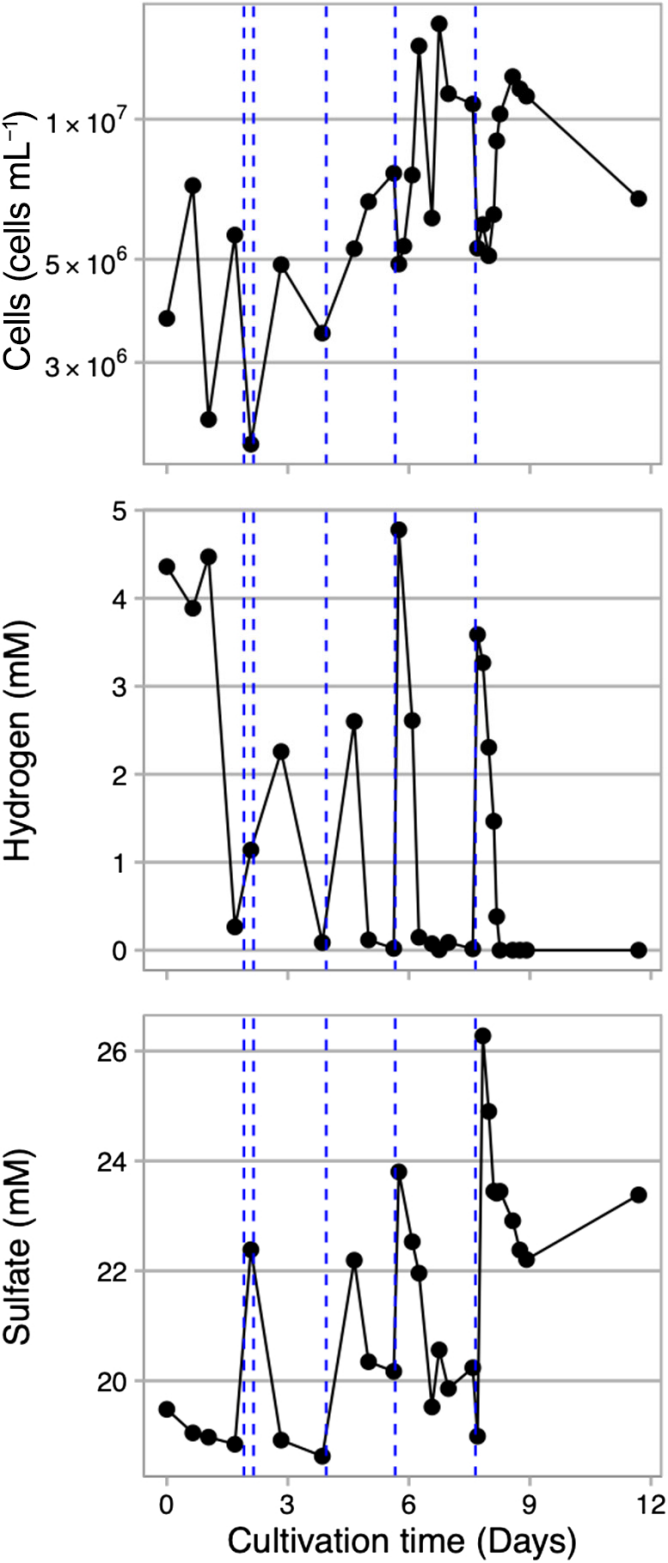
Growth curve of *Archaeoglobus profundus* AV18^T^ and environmental parameters during a cultivation experiment at 20 MPa and 82°C in the HTP-KCC system. The blue dotted line represents the time the hydrogen-dissolved medium is added.

In the cultivation experiment of *A. profundus* AV18^T^, we repeatedly introduced hydrogen-containing medium into the incubation chamber using an HP syringe. Hydrogen initially added at a concentration of 4.5‍ ‍mM was rapidly consumed by *A. profundus* AV18^T^ (Fig. 3). To sustain growth, medium containing hydrogen and sulfate was supplemented five times over the 11-day cultivation period. Within a few days after medium addition, cell growth was repeatedly activated and cell concentrations reached up to 1.6×10^7^‍ ‍cells‍ ‍mL^–1^. Since the inner incubation chamber has a volume of approximately 60‍ ‍mL, repeated sampling reduced the volume of culture medium, necessitating the supply of fresh medium for continuing long-term culture experiments with sampling. We maintained the culture of *A. profundus* AV18^T^ for up to 37 days by continuously supplying hydrogen-dissolved medium (Supplementary Fig. S4).

These experiments demonstrated the successful cultivation of an obligate hyperthermophilic piezophile and hydrogenotrophic hyperthermophile under anaerobic conditions, with periodic sampling being performed without disturbing high-pressure, high-temperature HHP-HT conditions.

### Effects of HHP changes at the time of inoculations on the growth of *P. horikoshii* OT3^T^

Even when a culture strain is isolated from the deep sea, it is typically cultured and stored at ambient pressure, except for obligate piezophiles. Although previous studies reported the negative effects of depressurization during the HHP cultivation of piezophilic cells (Cario *et al.*, 2022), the effects of an initial change in pressure from the ambient pressure-inoculum to the HHP culture, particularly on the early stages of the growth of piezophiles, remain unclear because of the limited availability of cultivation systems allowing isobaric inoculation. To adapt to the significant increase in pressure, it may be necessary for inoculated piezophiles to undergo physiological changes during the early stages of growth. Therefore, we hypothesized that cell physiology may differ between ambient pressure- and HHP-prepared inocula and conducted a comparative experiment using the hyperthermophilic archaeon *P. horikoshii* OT3^T^.

**Fig. 4.**
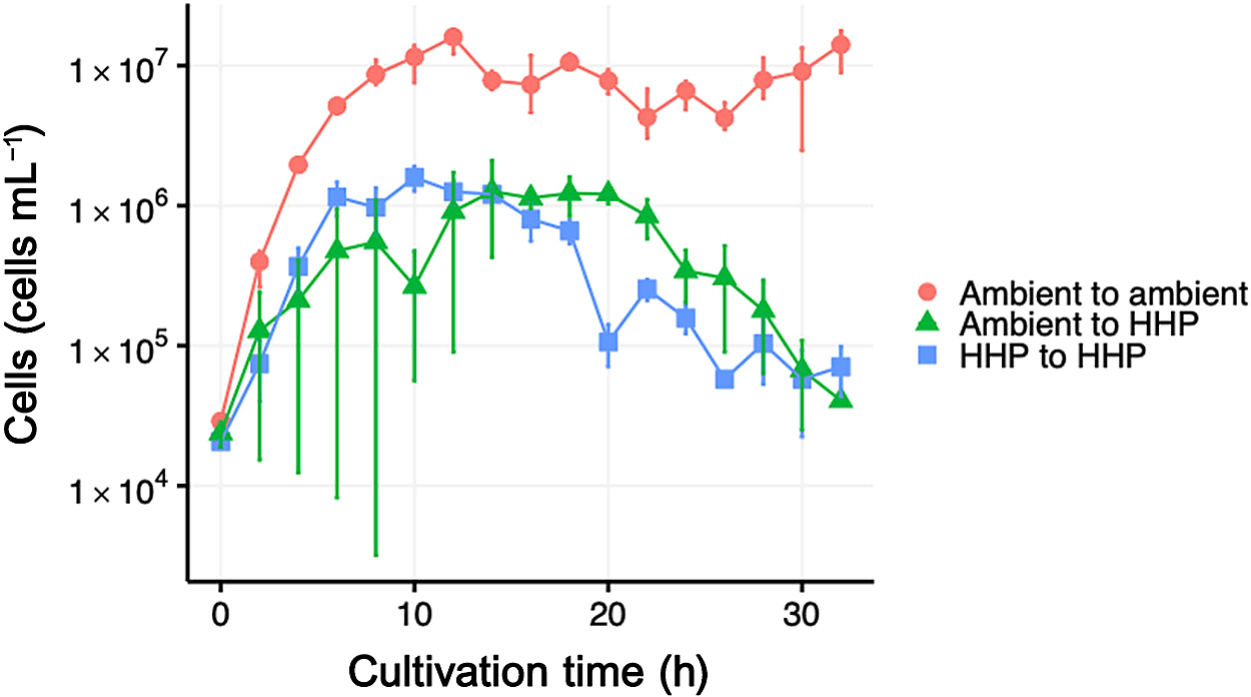
Growth curve of *Pyrococcus horikoshii* OT3^T^ during the cultivation experiment. Error bars represent the cell abundance range from triplicate experiments for “ambient to ambient” and a duplicate for “ambient to HHP” and “HHP to HHP”.

We conducted cultivation experiments on *P. horikoshii* OT3^T^ using inocula cultured under ambient pressure or HHP conditions. Three different combinations of inoculum and cultivation conditions were tested: (i) ambient to ambient, (ii) ambient to HHP, and (iii) HHP to HHP. The resulting growth curves of *P. horikoshii* OT3^T^ are shown in Fig. 4. A marked difference was observed in the maximum reachable cell concentration between the ambient and HHP cultivations. The number of *P. horikoshii* OT3^T^ cultured under ambient pressure increased to 1.6–1.8×10^7^‍ ‍cells‍ ‍mL^–1^, whereas those cultured under HHP conditions only increased to 1.0–2.1×10^6^‍ ‍cells‍ ‍mL^–1^. Similarly, the highest specific growth rate of 40‍ ‍min^–1^ was observed for ambient pressure cultivation. The effects of HT-HHP conditions on the growth characteristics of *P. horikoshii* OT3^T^ were investigated under a variable range of temperature (90–105°C) and pressure (0.1–60 MPa) conditions (Kato, 2006). Kato showed a piezophilic increase in the specific growth rate with elevations in pressure at higher temperature incubation conditions >100°C. However, at our incubation temperature of 98°C, we observed a maximum specific growth rate decrease in the HHP incubation. Although it is challenging to make direct comparisons due to the lack of experimental details described in Kato (2006), a 2°C difference may have contributed to the decline in the specific growth rate observed in our incubations. In cultivations of the ambient inoculum to HHP, we obtained variable growth outcomes depending on the trial. This variation is evident from the large error bars in Fig. 4 (individual growth curves and an additional cultivation experiment result are shown in supplementary Fig. S5). This variability was only observed under the “ambient to HHP” condition where a pressure transition occurred upon the inoculation. Occasionally, the “ambient to HHP” transition caused an initial decrease in the cell concentration, which we did not observe in the “ambient to ambient” and “HHP to HHP” conditions. Furthermore, the “ambient to HHP” condition sometimes resulted in no growth of the culture (data not shown). Based on the results obtained under the “ambient to HHP” condition only, we speculated that this may be caused by cellular responses that require a physiological shift in *P. horikoshii* OT3^T^ for adaptation from the “ambient to high pressure” condition.

In contrast, minimal variations and a faster growth rate were observed during the HHP to HHP cultivation, further supporting this notion. To the best of our knowledge, the present study is the first precise examination of the initial effects of a marked change in hydrostatic pressure on the physiology of hyperthermophilic archaeon. Our results underscore the importance of maintaining a constant pressure condition for successfully investigating piezophile physiology, particularly in high-temperature environments.

### Conclusion

We developed a novel HT-HHP cultivation system called HTP-KCC, which offers unique capabilities for handling pure cultures and environmental samples. This system enables us to perform incubation experiments without terminating the experiment for sample injection or sampling. In pilot studies, we successfully cultivated both piezophilic and H_2_-utilizing hyperthermophiles. A key feature of the HTP-KCC system is the ability to inoculate medium under isobaric conditions at HT-HHP, thereby allowing the precise control of the experimental environment and immediate monitoring of microbial cultivation progress from the start of the incubation experiment. This distinctive characteristic allowed us to observe the initial phase of *P. horikoshii* OT3^T^ cultivation as well as the negative impact of pressure changes on the physiology of piezophiles. While further investigations are needed, the present results demonstrate the significance of comprehensive monitoring in HT-HHP cultivations and the precise control of experimental conditions. With the HTP-KCC system, we aspire to elucidate the unexplored physiology of hyperthermophilic and/or piezophilic microorganisms in future endeavors.

## Citation

Mori, F., Ijiri, A., Nishimura, T., Wakamatsu, T., Katsuki, N., and Morono, Y. (2023) Cultivation of Piezotolerant and Piezophilic Hyperthermophiles with a Newly Developed Constant High Pressure and Temperature Culturing and Monitoring System. *Microbes Environ ***38**: ME23055.

https://doi.org/10.1264/jsme2.ME23055

## Supplementary Material

Supplementary Material
