# Supplementary material for: Cultivation of Piezotolerant and Piezophilic Hyperthermophiles with a Newly Developed Constant High Pressure and Temperature Culturing and Monitoring System
Source: Microbes Environ. 2023 Oct 20;38(6):ME23055. doi: 10.1264/jsme2.ME23055 (PMC10728627; doi:10.1264/jsme2.ME23055)
Supplement: Supplementary file 1 — Supplementary Material [file 38_23055_s1.pdf]

Supplementary materials for

Cultivation of Piezotolerant and Piezophilic Hyperthermophiles with a Newly Developed Constant High Pressure and Temperature Culturing and Monitoring System

Fumiaki Mori[1], Akira Ijiri[1, 2], Tomoya Nishimura[1,3], Taisuke Wakamatsu[4], Nozomi Katsuki[4, 5], and Yuki Morono[1*]

[1] Geomicrobiology Group, Kochi Institute for Core Sample Research, Extra-cutting-edge Science and Technology Avant-garde Research (X-star), Japan Agency for Earth-Marine Science and Technology (JAMSTEC), Monobe B200, Nankoku, Kochi 783-8502, Japan

[2]Graduate School of Maritime Sciences, Kobe University, 5-1-1 Fukaeminamimachi, Higashinada-ku, Kobe 658-0022, Japan

[3]Applied Science, Graduate School of Integrated Arts and Sciences, Kochi University, Monobe B200, Nankoku, Kochi 783-8502, Japan

[4]Agricultural Science, Graduate School of Integrated Arts and Sciences, Kochi University, Monobe B200, Nankoku, Kochi 783-8502, Japan

[5] Faculty of Life and Environmental Sciences, University of Tsukuba, 1-1-1 Tennodai, Tsukuba, Ibaraki 305-8572, Japan.

*** Corresponding Author.** Email: morono@jamstec.go.jp; Tel +81-88-878-2273

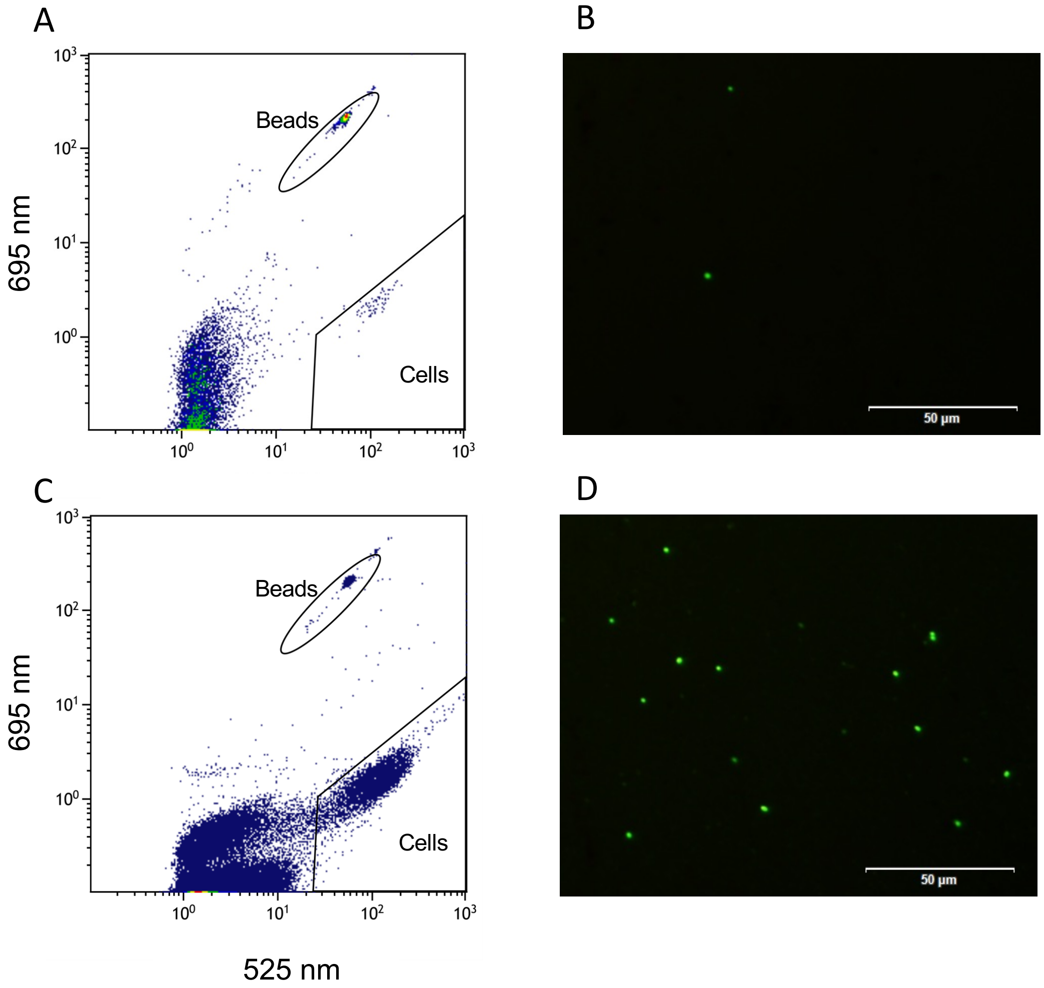

Fig. S1 Flow cytograms (A and C) and microscopic photography (B and D) of *P. yayanosii* CH1^T incubated for 2 (A and B) and 30 hours (C and D) at 98°C and 52 MPa in the HTP-KCC. The lower right portion below the solid line shows the region of the cell and the circle indicates the dots of beads in (A) and (C). All cytograms show approximately 1,800 dots of beads. The sample was stained using SYBR Green I.

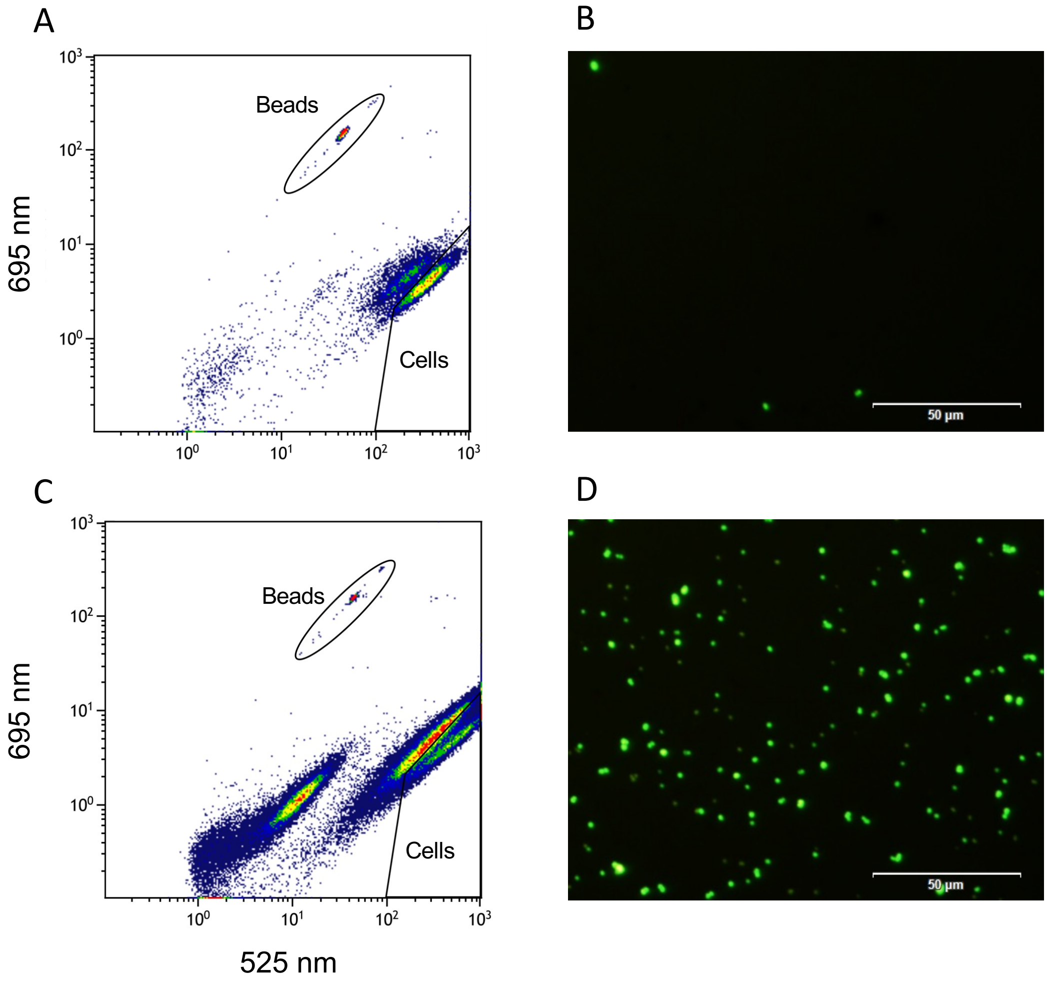

Fig. S2 Flow cytograms (A and C) and microscopic photography (B and D) of *P. horikoshii* OT3^{T} incubated for 2 (A and B) and 32 hours (C and D) at ambient pressure. The lower right portion below the solid line shows the region of the cell and the circle indicates the dots of beads in (A) and (C). All cytograms show approximately 2,000 dots of beads. The sample was stained using SYBR Green I.

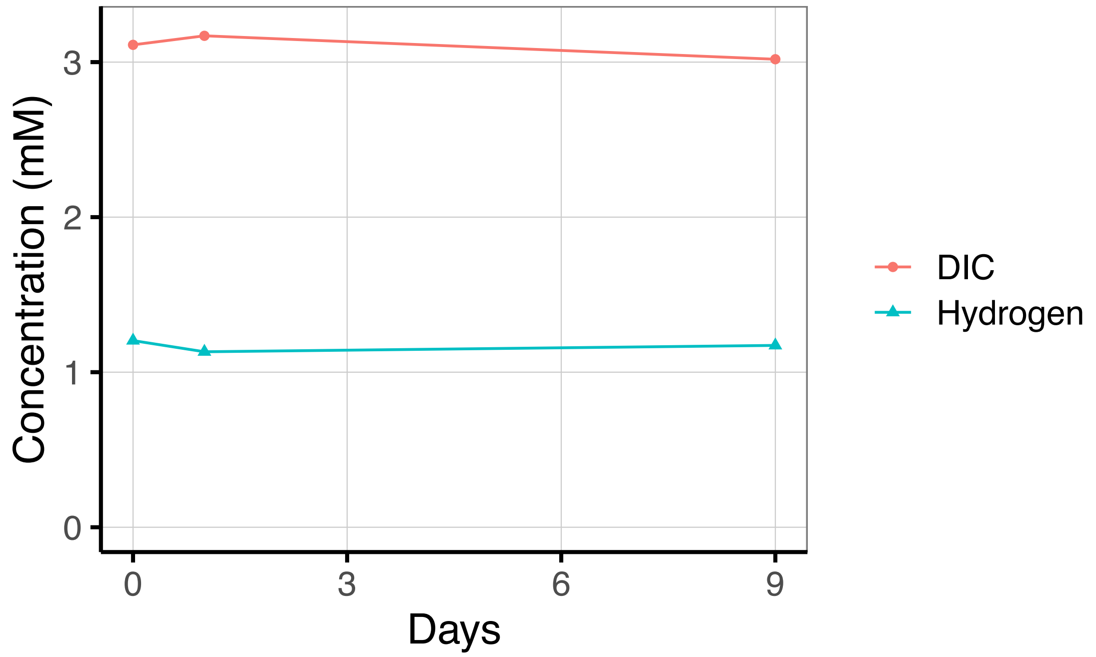

Fig. S3 Concentration of hydrogen and DIC in the medium that was incubated at 82°C and 20 MPa in the HTP-KCC system.

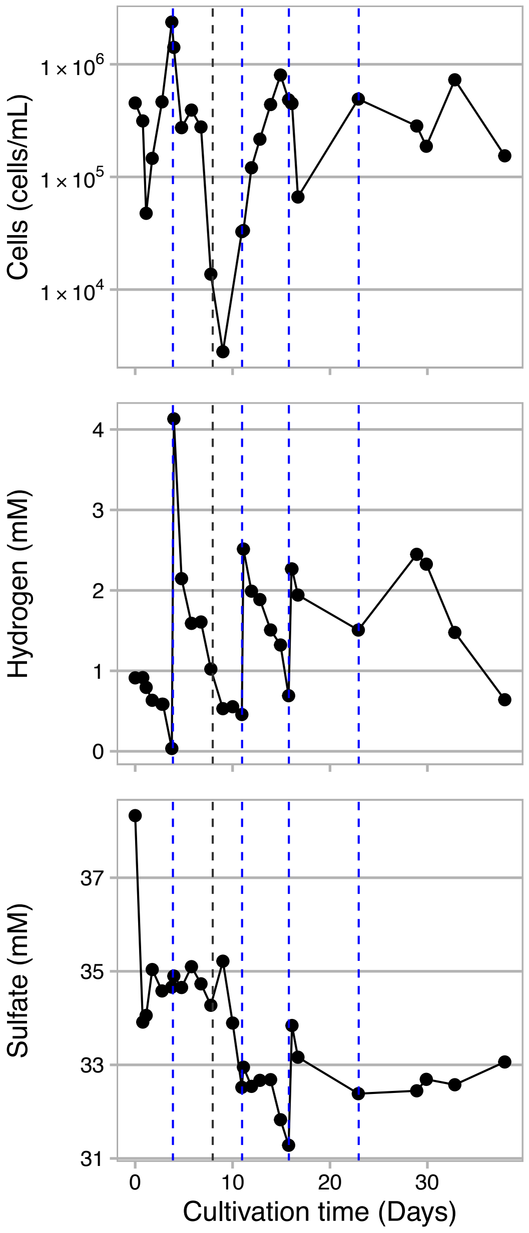

Fig. S4 Growth curve of *A. profundus* AV18^T and environmental parameters during cultivation experiment at 20 MPa and 82°C in the HTP-KCC system. The blue and gray dotted lines represent the time of addition of hydrogen-dissolved and non-hydrogen-dissolved medium, respectively.

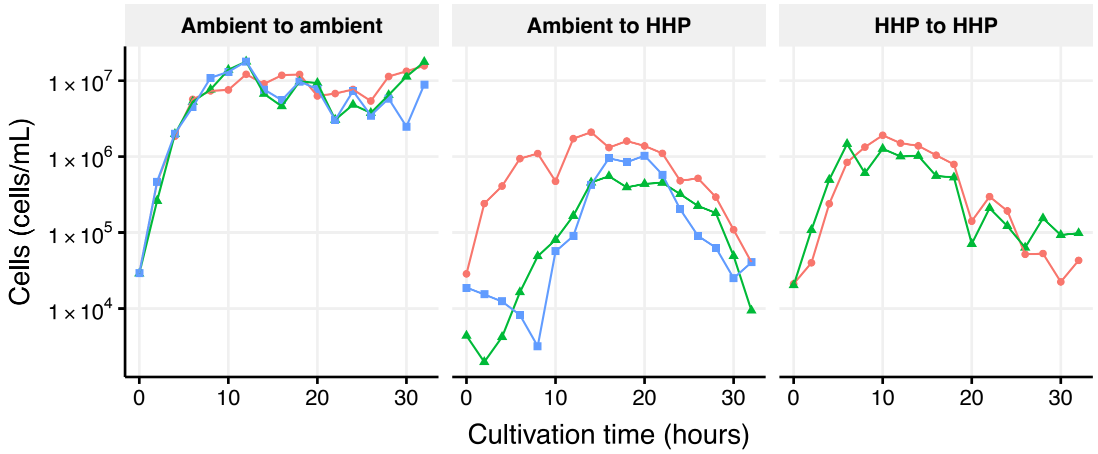

Fig. S5 Growth curve of *P. horikoshii* OT3^{T} during cultivation experiment. An additional result of the "Ambient to HHP" cultivation experiment was also shown (triangle and green line in the "Ambient to HHP" figure) and also showed a decrease in cell abundance during the early stages of culture.
